# "The Hospital" Commission and Beer and Stout

**Published:** 1909-08-14

**Authors:** 


### The Hospital

A Journal of

The Medical Sciences and Hospital Administration.

New Series. No. 129, Vol. V. [No. 1201, Vol. XLVI.] SATURDAY, AUGUST 14, 1909.

### "The Hospital" Commission and Beer and Stout.

We have received a copy of "The Literary Digest" containing an article entitled "More about the Nutritive Value of Beer." This article consists essentially of extracts from two other articles criticising the report of The Hospital Commission upon beer and stout. Each author supports his criticisms by weighty quotations from the writings of physiological chemists. These quotations are for the most part in themselves accurate and true, but robbed of their context and flung haphazard at what the authors assume to be the teaching of The Hospital Commissioners' report, they can only be described as irrelevant and absurd. The critics above referred to seem to be under the extraordinary impression that our Commissioners recommended beer and stout as a sole article of diet, and thus as a sole source of the energy required by the working individual. It is quite obvious that deductions based upon such a hypothesis would lead only to dietetic results as grotesque as they would be impossible. In their eager fight against this thesis, which they themselves have manufactured purely for the sake of virulent anti-alcoholic polemics, they seem to have entirely lost sight of the main teaching of The Hospital's report, which essentially was that good beer was a good drink, assuaging thirst, and at the same time supplying nutriment to the body, quite apart from the alcohol it contained. We entirely disagree that any of the authorities named have proved that beer or stout in ordinary dietetic quantity has any injurious effects either *qua* its alcohol or its other constituents, upon the digestive processes *in vivo.* There are some individuals with whom malt liquors do not agree, just as there are others to whom tea is poison, and in whom milk causes marked gastric disturbance. Dr. Kellog is quite mistaken in supposing that our Commissioners recommended the consumption of a gallon of beer a day, or, indeed, that any of the calories required in ordinary life should be derived from beer. What we did say was that of alcoholic beverages beer and stout were the most nutritive, and that they contained nutriment quite apart from their alcohol. Nobody in their right senses would recommend milk as a sole article of diet to a working individual, although an occasional glass of milk may make an exceedingly useful addition to many dietaries. Beer is essentially a drink, and is primarily taken for the quenching of thirst; but at the same time those who take beer in dietetic quantities add materially to the calorimetric value of their original diet. We entirely disagree with regard to the indigestibility of the nutritive elements in beer; our observation and experience tell us that the carbohydrates of beer are exceedingly well borne and easily digested by the average individual. As an instance we may mention the value of good stout in women during the period of suckling. We may further remark that our article was not primarily intended for the public, but for the medical profession, before whom we wished to place fact. It is for the medical man who knows the individual to indicate whether to give beer or not, and should he feel justified in recommending beer, the kind to recommend. While we admire the philanthropic élan of our critics, we regret that they should have entirely misinterpreted our report. We have the philanthropist's desire to improve mankind, but this is tempered with the scientist's regard for fact. Whether beer does more moral harm than it does physical good is not the problem we, as a scientific journal, set ourselves to answer. Our problem was to see actually what the ordinary beer was, to discriminate between good and bad, and, finally, to indicate in what way beer could be used for the physical good of the individual. Human beings have to drink something, and the mere fact that a man likes a certain drink is presumptive evidence that that drink is good for him. In beer and stout we have drinks of varying but, generally speaking, low alcoholic strength, and drinks in which the alcohol is by no means the most essential factor. This quality of beer and stout at once differentiate them from distilled spirits, which are practically alcohol pure and simple and can never be made into anything more than dilute alcohol. We entirely object, from the scientific standpoint, to the thesis that because a beverage contains alcohol it is *ipso facto* injurious to health. As we have frequently pointed out elsewhere, many so-called teetotal drinks contain percentages of alcohol of the same order of magnitude as those of beer. The excise authorities of this country rightly recognise that alcohol must occur as a by-product in the manufacture of certain beverages, and therefore exempt from duty any beverage containing less than 2 per cent. of proof-spirit. The difference between a drink containing 2 per cent. of proof-spirit and one containing 4 per cent. cannot be that between a harmless beverage and a poison, and to speak of light and natural alcoholic beverages which have been consumed by the majority of mankind harmlessly for centuries as poisons, is in our view both unscientific and misleading. Violent and inaccurate terminology of this type will never influence those whose influence is worth obtaining. The real problem for temperance reformers, who have our sincerest sympathy and can always rely upon our constant help, is not to inveigh by such means against every and all class of alcoholic drink, but to teach the public, especially the young, as is being done so conscientiously in France, not only how not to mis-use but also how to use alcoholic beverage. We must conclude this reply to our critics by repeating the words of our report, which really contain the gist of the whole matter, and which escaped criticism because they are indisputable—namely that "beer and stout of proper quality and given in proper doses and in suitable cases, will be found amongst the most useful dietetic and, indeed, medicinal agencies we possess."

